# Beware of the structural heterogeneity among SIRPB1 isoforms

**DOI:** 10.1186/s12967-024-05365-7

**Published:** 2024-09-09

**Authors:** E Alarcón-Martín, MJ Bravo, JL Royo

**Affiliations:** 1 Departamento de Especialidades Quirúrgicas, Bioquímica e Inmunología, Facultad de Medicina, University of Malaga, Málaga, Spain; 2 Research Center and Memory Clinic, ACE Alzheimer Center Barcelona, Universitat Internacional de Catalunya, Barcelona, Spain

To the Editor,

It has been recently published that the *SIRPB1* expression is upregulated in gliomas, which had an adverse effect on the immune milieu and correlated poorly with patient survival [1]. Geng and coworkers show SIRPB1 activation by a specific antibody results in SYK phosphorylation and subsequent activation of the signaling pathway. Co-culture experiments demonstrated that macrophages with *SIRPB1* knockout showed decreased IL1RA, CCL2, and IL-8, which were recovered upon ectopic expression of *SIRPB1* but reduced again following treatment with SYK inhibitor. Critically, lower overall survival rates were associated to increased SIRPB1 expression. This is not the first time that the role of SIRPB1 has been analyzed in the context of solid tumors. In prostate cancer, inhibiting SIRPB1 lowers CDK4 expression, which reduced prostate cancer cell proliferation by arresting the cell cycle in cell xenografts [2]. In these cells, SIRPB1 downstream pathway was associated to increasing AKT phosphorylation what was found crucial for prostate cancer cell proliferation. In addition, it has also been reported that SIRPB1 participates in breast cancer progression [3]. Chen and coworkers reported that the engulfment of mesenchymal stem cells by cancer cells leads to significant changes in gene expression profiles of breast cancer cells, with an upregulation of genes encoding extracellular and cell surface proteins including SIRPB1. In the context of the interaction between breast cancer cells and mesenchymal stem cells, SIRPB1 could be involved in promoting invasion and metastasis by facilitating the interaction between cancer cells and mesenchymal stem cells in the tumor microenvironment.

All these studies refer to SIRPB1 as a 398 aa-long membrane protein with an apparent weight of 55 kDa. However recent genetic and molecular studies reveal that the *SIRPB1 locus* contains a common copy-number variation (CNV), represented by a frequent (≈ 20%, minor allele frequency) insertion found in all human populations. The insertion allele changes the SIRPB1 protein structure leading to four different protein-coding isoforms [4]. This implies that approximately 36% of the general population contains at least one copy of the insertion allele and therefore their immune cells express not only the most common one, named isoform 205, but also isoforms 201, 202 and 204 depending on their specific genotype (Table 1).

**Table 1:** Description of the SIRPB1 isoforms

SIRPB1 Isoform	Alternative name	Length	UniProt	SIRPB1 CNV genotype		
				Del/Del	Ins/Del	Ins/Ins
201		180	H9KV29	NO	YES	YES
202	213	398	Q5TFQ8-1	NO	YES	YES
204		181	O00241-2	YES	YES	NO
205		398	O00241-1	YES	YES	YES

The table presents a brief description of the SIRPB1 isoforms. Only those leading to full-length protein-coding isoforms were include. The corresponding UniProt code is provided. SIRPB1 copy-number genotype is illustrated as Deletion (Del) or insertion (Ins)

Differences among these isoforms are not minor and affect both gene expression and protein structure. Genotype-Tissue Expression (GTEx) data available for SIRPB1 shows that isoform 205 exhibits the highest expression levels, followed by 204 and finally 201. Isoform 202 shows minimal expression in any tissue. However, isoform 213 shares the same resulting protein with different messenger RNA and exhibits slightly more ubiquitous expression (Fig. 1).

**Fig. 1:**
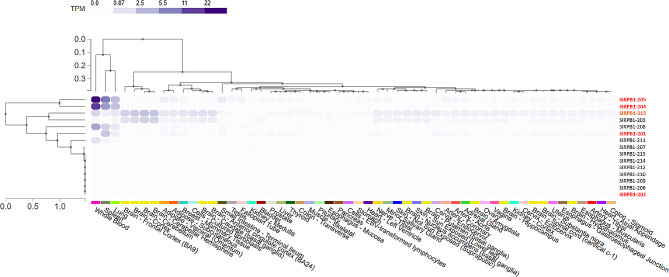
Heatmap generated from GTEx data, illustrating the tissue expression of RNA from SIRPB1 gene isoforms. The isoforms selected for this study due to their ability to encode proteins are highlighted in red. Additionally, isoforms preselected that share amino acid sequences with the isoforms highlighted in red are marked in orange

From the protein-structure point of view, we also find differences among their structural domains. Interpro analysis revealed that all SIRPB1 isoforms feature a signal peptide, concordant with their membrane subcellular localization of all isoforms. However, they differ in the number of Ig-like domains: one for SIRPB1-201 and − 204, while three for SIRPB1-202 and − 205. This also implicates that the western-blot analysis available in the literature showing a unique 55 kDa band is only compatible with isoforms 202 and 205 (Fig. 2), while > 35% of the population also express isoforms 201 and 204.

**Fig. 2:**
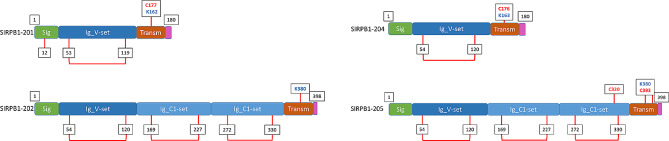
The image displays the different domains of SIRPB1 isoforms. In green, we have the signal peptide; in dark blue, the Ig-LikeV domain; in light blue, the Ig-LikeC domain; in brown, the transmembrane domain; and in pink, the cytosolic domain. The values below mark the cysteines that are not relevant for dimerization, either because they are in the signal peptide or because they form a disulfide bridge (marked with a red connection between two values). The remaining cysteines available for potential dimerization are marked in red, as well as the charged lysines of the transmembrane domain

These structural differences are crucial as they may be involved in the protein’s function and their implication in different pathologies. The extracellular domains are responsible for interacting with potential ligands or associated proteins. We have recently described that isoforms 201 and 205 are capable of inducing beta-amyloid phagocytosis via DAP12 in transfected HEK293 cells, while isoforms 202 and 204 do not. The ligands in a solid tumor context still remain elusive. Regarding dimerization, we should highlight that Liu and coworkers reported the effect of point mutation assays, what defined SIRPB1 as a homodimer, with cysteine 320 being key to the structure [4]. However, only isoform 205 contains this cysteine, which suggests that the other three isoforms might be functional as monomers. Finally, we have to take into account that SIRPB1 does not contain an intracellular domain, which turns its transmembrane domain as a crucial way of transducing the extracellular signal. Again, we find here a key difference between isoforms. While isoforms 201, 204, and 205 share the same transmembrane domain, isoform 202 has an alternative sequence. When theie binding affinity for DAP12 transmembrane domain was evaluated in vitro, we found that isoform 204 transmembrane domain has a lower affinity for DAP12 [5]. With all these data in mind, a detail evaluation of the technical aspects of the literature, including the last paper described by Geng and coworkers [1], showed that the conclusions are based on just one of these isoforms: SIRPB1-205. This does not invalidate Geng’s team data, but might be limiting our comprehension of the true role of SIRPB1 in solid tumor progression. Thus, future studies shall consider the SIRPB1 protein structure heterogeneity to determine which isoform/s are implicated in the different phenotypes studied so far.

## Data Availability

All data for this study are publicly available.
